# Is (critical) health literacy a key to better psychosomatic functioning in patients with inflammatory bowel disease? Testing a mediation model

**DOI:** 10.3389/fpsyt.2026.1643641

**Published:** 2026-02-06

**Authors:** Orsolya Papp-Zipernovszky, Barbara Horvát, Anett Dávid, Beatrix Rafael, Sanela Tóth-Njers, Dávid Strausz, Mátyás Gyóllai, Tamás Molnár, Viola Sallay, Barna Konkolÿ Thege, Tamás Martos

**Affiliations:** 1 Department of Personality and Health Psychology, Institute of Psychology, Eötvös Loránd University, Budapest, Hungary; 2 Heart and Vascular Center, Faculty of Medicine, Semmelweis University, Budapest, Hungary; 3 Department of Personality, Clinical and Health Psychology, Institute of Psychology, University of Szeged, Szeged, Hungary; 4 Department of Internal Medicine, University of Szeged, Szeged, Hungary; 5 Department of Preventive Medicine, University of Szeged, Szeged, Hungary; 6 Department of Cognitive and Neuropsychology, Institute of Psychology, University of Szeged, Szeged, Hungary; 7 Student, University of Szeged, Szeged, Hungary; 8 Paris Department of the Faculty for Psychotherapy Science, Sigmund Freud Private University, Vienna, Austria; 9 Waypoint Research Institute, Waypoint Centre for Mental Health Care, Penetanguishene, ON, Canada; 10 Department of Psychiatry, University of Toronto, Toronto, ON, Canada

**Keywords:** depression, health literacy, health self-efficacy, inflammatory bowel disease, patient-reported outcomes, structural equation modeling

## Abstract

**Introduction:**

Chronic illnesses such as inflammatory bowel disease (IBD) require continuous self-management, often under emotionally and physically taxing conditions. While health literacy and health self-efficacy are known to support disease adaptation, their combined role in psychosomatic functioning, especially under varying levels of depression, remains underexplored. This study examined how health literacy, health self-efficacy, and depressive symptoms influence symptom severity and life satisfaction in patients with IBD.

**Methods:**

A cross-sectional survey of 393 patients with IBD (60.7% with Crohn’s disease; 56% female; mean age = 40.75) was conducted at a gastroenterology outpatient clinic in Hungary. Standardized questionnaires assessed health literacy, health self-efficacy, depression, symptom severity, and satisfaction with life. Structural equation modeling was used to test a mediation model. Multigroup analyses explored the stability of the model across subgroups defined by depressive symptom levels, disease status (relapse vs. remission), and types of diseases.

**Results:**

Critical health literacy predicted higher health self-efficacy, which was associated with lower symptom severity and, in turn, greater life satisfaction. This indirect pathway remained significant after controlling demographic variables. Multigroup analyses showed that these relationships were stronger among patients in relapse and those with elevated depression, suggesting increased psychological sensitivity in these subgroups. No difference was found between types of disease.

**Discussion:**

The findings underscore the importance of critical health literacy and health self-efficacy as interconnected psychological resources in chronic illness self-management. Strengthening these capacities may reduce symptom burden and enhance well-being, particularly in times of relapse and periods of psychological vulnerability. The results support a shift toward integrated, psychosocially informed care models for IBD.

## 1 Introduction

Inflammatory bowel disease is an umbrella term for two types of immune-mediated diseases of the gastrointestinal tract with very similar pathophysiology, such as ulcerative colitis and Crohn’s disease (1). The common feature of these diseases is the chronic relapse of inflammation of the intestinal tract, which may manifest itself in stomach pain, abscesses in the gastrointestinal system, bleeding and anemia, diarrhea and vomiting, various malabsorption disorders, weight loss, fatigue, and even arthritic symptoms (2, 3). During periods of remission, these symptoms significantly improve or even disappear (4).

The development of inflammatory bowel diseases is thought to be complex and multifactorial: alterations in genes responsible for regulating the immune response (5), environmental factors (e.g., pollution), and risk behaviors, including inadequate diet, drug use, vitamin D deficiency, and, especially in Crohn’s disease, smoking (6). Chronic psychosocial stress has also been shown to affect the occurrence of relapses and may, therefore, worsen the progression of the disease (7).

Several studies pointed out the burden of inflammatory bowel diseases on mental health and (health-related) quality of life (8). Studies focusing on anxiety and depressive disorders among patients with inflammatory bowel disease highlighted that the prevalence of these mental disorders is higher compared to the general population (9–13) or populations with other chronic diseases (14–16). Kochar and colleagues (17) found a clear correlation between the worsening of physical symptoms in inflammatory bowel disease and the increase in depression score in twenty-two months of living with the disease. Furthermore, Nahon and colleagues (18) and Mardini and colleagues (19) found evidence for the interconnection of the worsening of depressive and anxiety symptoms and disease relapses.

Management of the disease requires lifelong adherence to medication, including more complex treatments such as enemas or self-injected biological therapies. Furthermore, dietary management, performing relaxation exercises, and learning to acknowledge the progression of the disease or the emergence of mental health problems are also critical in coping with the disease (20). Horvat and colleagues (21) conducted a multigroup analysis among patients with inflammatory bowel disease in remission and in relapse to investigate the differences in their self-management. The authors identified separate patterns that considered the characteristics of health goals and related emotions during the two stages of the disease. Specifically, patients in relapse were more sensitive toward the quality of received support, and their negative emotional experiences were more indicative of lower life satisfaction than in patients in remission.

According to an Australian study, patients with inflammatory bowel disease would require more information from their health professionals about medications and their side effects, diet, disease complications, and how to access specialized nurses (22). These all require acquiring new information and skills to navigate the healthcare system, which is called health literacy (23). Nutbeam (24) proposed a three-level health literacy framework that includes (a) functional health literacy, comprising of skills for reading, writing and counting that are applied in everyday situations; (b) communicative health literacy, referring to more advanced skills in seeking information from different (interactive) sources, and actively using them in changing environments; and (c) critical health literacy, including the most developed skills for critically analyzing information and applying them to gain greater control over someone’s health and to contribute to optimizing health services.

To achieve balanced chronic disease management and acceptable health outcomes, patients also need a belief that they can cope with the newly emerging difficulties, which is called self-efficacy (25). Previous studies have pointed out that health-related self-efficacy is a relevant component in disease management and coping among patients with inflammatory bowel disease (26, 27). In a study examining patients with type 2 diabetes mellitus (T2DM), lower self-efficacy was found in the group with high depression (2-item Patient Health Questionnaire scored ≥ 2). In a further logistic regression analysis, those who were depressed displayed over two times higher likelihood of low health literacy. Finally, high depression and low disease knowledge were both significant, independent predictors of low diabetes self-management (28). However, the role of depression, health literacy, self-efficacy, and other mediatory variables influencing clinical outcomes in inflammatory bowel disease is poorly understood (3).

One of the first studies including patients with inflammatory bowel disease that used a more complex model by measuring health literacy, health self-efficacy, quality of life, depression, and clinical disease activity was carried out by Tormey and colleagues (29). A shortcoming of their work was the low number of enrolled participants (n = 99). However, the authors drew attention to the considerable proportion of patients with inflammatory bowel disease possessing limited health literacy (40%), as well as the consequences of low health literacy, such as worse quality of life, lower subjective health status, and more depressive symptoms. Dos Santos Marques and colleagues (3) found a lower percentage of limited health literacy, 24%, among 175 patients with inflammatory bowel disease and measured almost the same patient-reported health outcomes (e.g., health-related quality of life, depression) as Tormey and colleagues (29). Both health literacy and health self-efficacy were significantly associated with subjective health status and depression. The authors raise the need for studies examining larger samples of patients with inflammatory bowel disease and using more complex statistical models to understand the role of mediating and moderating variables between health literacy and subjective physical and psychological health outcomes (29). Hu and Xu (30) attempted to identify the relationship between fear of progression and health-related quality of life in patients with inflammatory bowel disease using sequential mediation analysis with the mediatory variables of health literacy and self-care. They found an unexpectedly high percentage (85.9%, 207 cases) of limited health literacy among patients with inflammatory bowel disease. Moreover, they confirmed a significant chain mediating effect of health literacy and self-care in the examined relationship.

### 1.2 The present study

Previous research suggests that health literacy, depression, and chronic disease are interconnected in several ways, influencing patient-reported outcomes (e.g., subjective health and satisfaction with life) of patients with inflammatory bowel disease through different mechanisms. In the present study, we aim a) to better understand the potential effect of health literacy on physical and mental health outcomes, b) to test the mediating role of health self-efficacy between health literacy and patient-reported, health-related outcomes, and c) to explore if these associations can be generalized across various background characteristics and health conditions such as depressive symptom severity, IBD status (remission vs. relapse), and type of disease (Crohn’s disease and colitis ulcerosa), in a larger sample of patients with inflammatory bowel disease.

**Figure 1:**
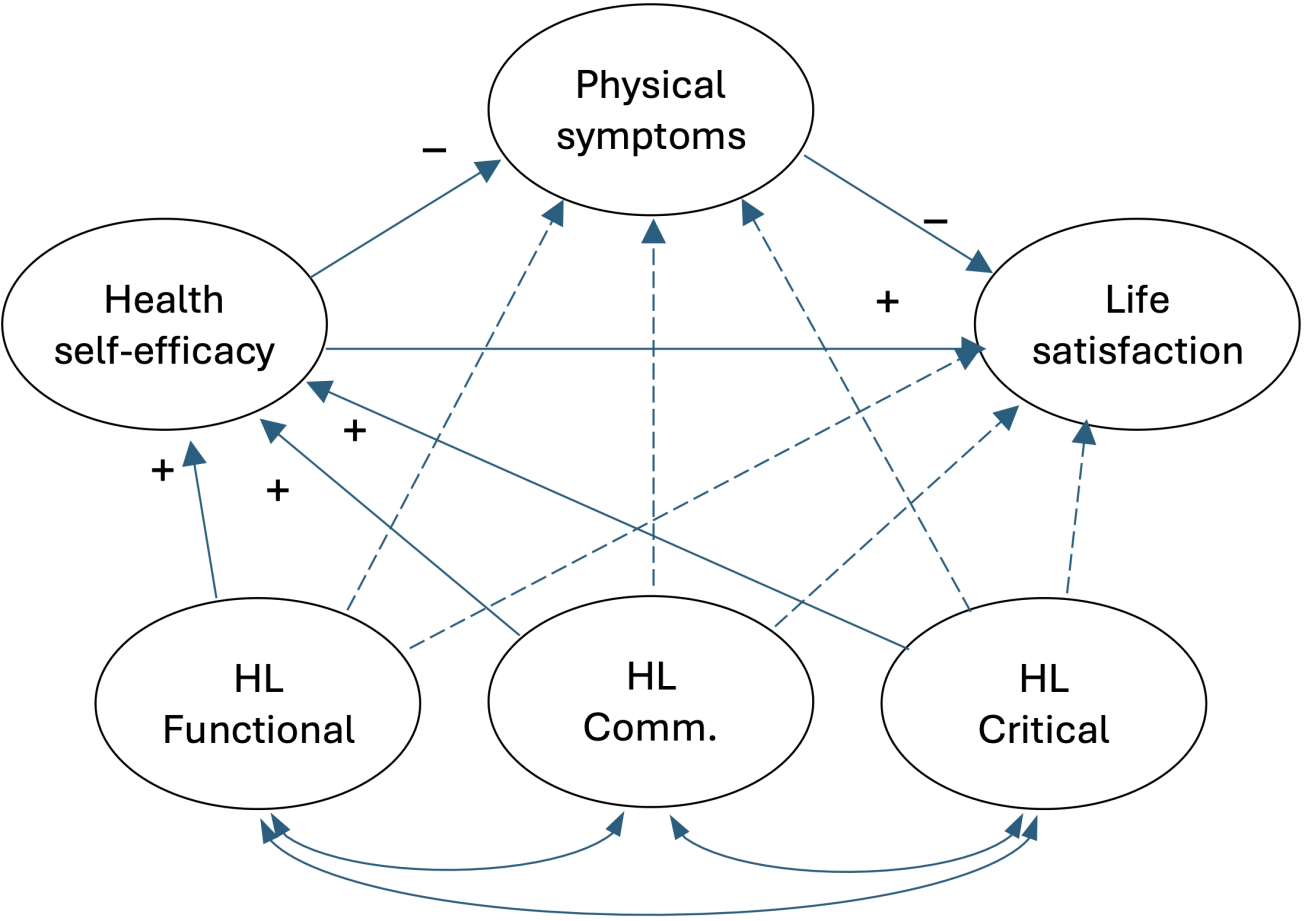
The conceptual model. HL, Health literacy; HL Comm., Communicative health literacy. Manifest variables are not included in the figure. Dashed lines represent paths that are tested for completeness but that are not central to our hypotheses.

First, based on the results of Hu and Xu (30), we propose a chain mediation model in which dimensions of health literacy predict health self-efficacy and patient-reported outcomes, such as physical symptom severity and life satisfaction (see **Figure 1** for further details). Specifically, we assume that higher health literacy predicts higher health self-efficacy, which in turn predicts lower symptom severity and higher life satisfaction in patients with inflammatory bowel disease. While we did not expect strong direct predictions from dimensions of health literacy to physical symptom severity and life satisfaction, we also tested these paths in the first model.

Second, we wanted to test whether the baseline model and its hypothesized associations hold after controlling for a series of sociodemographic characteristics, such as gender, age, level of education, and relationship status, that were found to be interrelated with health literacy (31) and other study variables.

Third, we wanted to explore whether the proposed mediation model was equivalent across subgroups of specific mental and physical health conditions. Considering the vulnerability of patients with inflammatory bowel disease to heightened depression and the bidirectional relation between health literacy and depression, we tested whether there was a difference in the fit of our model in patients with inflammatory bowel disease with and without heightened levels of depressive symptoms. Similarly, extending the findings of Horvat and colleagues (21) regarding the self-management of inflammatory bowel disease patients during relapse and remission, we also wanted to compare our theoretical model between these subgroups of patients. Finally, we also tested whether subgroups with two types of IBD, that is, Crohn’s disease and colitis ulcerosa, differ in their patterns of associations between the studied phenomena.

## 2 Methods

### 2.1 Participants and data collection procedure

The data were collected in frames of the research program “Health goals in a social ecological context: developing a Personal Niche Model of Health”. In sum, 417 patients with IBD (235 female, 182 male; mean age 41.5 years, SD = 12.0) were enrolled between November 2022 and January 2023 at the Gastrointestinal Outpatient Clinic of Internal Medicine of Szent-Györgyi Albert Clinical Center in Szeged, Hungary. The inclusion criteria were a diagnosis of inflammatory bowel disease and an age of over 18 years. The largest proportion of respondents (45.8%, n=191) completed secondary education and were diagnosed with Crohn’s disease (64.7%, n=255; n=23 missing). **Table 1** describes the sociodemographic data in detail.

**Table 1:** Demographic characteristics of the sample.

Categorical variables	N	%
Gender		
Female	235	65.0
Male	182	35.0
Level of education		
primary education	101	24.2
secondary education	191	45.8
higher education	125	30.0
Cohabitation		
Yes	297	71.2
No	109	26.1
missing	11	2.7
State of disease (self-record)		
Remission	278	66.7
Relapse	81	19.4
missing	58	13.9
Disease type (self-record)		
Crohn's disease	255	61.1
Colitis Ulcerosa	139	33.3
missing	23	5.5
Continuous variables	m	SD
Age (years)	41.5	12.0
Time passed since the onset of illness (years)	13.7	9.4
Subjective financial status (1-10)	5.4	1.7

Respondents were asked to complete a paper questionnaire pack voluntarily after being informed about the study. The research was carried out with the approval of the Regional Research Ethics Committee of the Albert Szent-Györgyi Health Center at the University of Szeged (180/2022-SZTE RKEB). This study was carried out according to the Code of Ethics of the World Medical Association (Declaration of Helsinki), and written informed consent was obtained from all participants. The complete questionnaire package took on average 25 minutes to complete.

### 2.2 Measures

Demographic characteristics assessed were age, educational attainment, gender, relationship status, and subjective financial status. We also inquired about the time passed since the onset of illness and the illness status (relapse or remission) as disease-specific characteristics.

Health literacy was assessed using the *Functional, Communicative and Critical Health Literacy Scale* (FCCHL, 32). The Hungarian version was validated in a general sample and evidenced good reliability and structural validity (Cronbach’s alphas ranging from 0.790 to 0.821; 31). The scale, consisting of 14 items, is divided into three subscales: the first five items refer to the degree of functional, items 6–10 to communicative, and items 11–14 to critical health literacy. Respondents can indicate their agreement with the statements on a four-point Likert scale (0 = never, 1 = rarely, 2 = sometimes, 3 = often). The measurement can also provide a summarized general health literacy score, with higher scores indicating a higher level of health literacy. For all scales used in this study, we estimated preliminary Cronbach’s alphas and omegas as reliability estimates in the sample. For each FCCHL subscale (α_Functional_ = 0.774, α_Communicative_ = 0.712, α_Critical_ = 0.844 and ω_Functional_ = 0.790, ω_Communicative_ = 0.727, ω_Critical_ = 0.847), internal consistency estimates were adequate, indicating that the subscales are appropriate for further analyses.

To assess health self-efficacy, the 4-item shortened Hungarian version of the *General Self-Efficacy Scale* (GSES, 33, 34) was adapted for health-related statements (e.g., “I can usually handle situations related to my health.”) and evidenced good reliability in a general sample (α = 0.82; 35). The items can be rated on a 5-point Likert scale (1 = not at all true; 5 = completely true). The test had good preliminary reliability in the present sample (α = 0.818; ω = 0.819).

Depression was measured using the three-item depression subscale of the shortened, 9-item Hungarian version of the *Depression, Anxiety, Stress Scale* (DASS, 36–38). The level of agreement with the statements could be indicated on a 4-point Likert scale with reference to the recent weeks (e.g., “I felt that nothing good in life was waiting for me.”; 0 = did not apply to me at all, 3 = very frequently applied to me). The depression subscale showed adequate reliability in previous studies (α = 0.751), whereby a higher score indicates the presence of more depressive symptoms. The internal reliability of the scale was found to be adequate in the present sample too (α = 0.746; ω = 0.760).

To account for patient-reported outcomes (PROs), we measured satisfaction with life, the presence and seriousness of physical symptoms of inflammatory bowel disease, and the state of remission or relapse.

Satisfaction with life was assessed using the Hungarian version (39) of Diener’s 5-item *Satisfaction with Life Scale* (40), which measures the cognitive, evaluative component of subjective life satisfaction. The validation of the Hungarian version across diverse subsamples provided evidence for the scale’s reliability (Cronbach’s alphas ranging from 0.84 to 0.89; 39) and validity. The agreement with the statements can be indicated on a 5-point Likert scale, where 1 = strongly disagree and 5 = strongly agree. The scale’s preliminary reliability was good (α = 0.859; ω = 0.860) in the present sample.

The presence and severity of physical symptoms were assessed using 14 items of the *Patient Health Questionnaire* (41), a self-report symptom checklist frequently used in Hungarian epidemiological surveys (42). The symptoms were stomach pain, back pain, arm/leg or joint pain, headache, chest pain, dizziness, fainting spells, heart palpitation, shortness of breath, pain or problems during sexual intercourse, constipation/loose bowels or diarrhea, nausea or indigestion, tiredness/having low energy, sleep problems, and chest pain. Patients indicated whether or not they had experienced each symptom in the last week (0 = no, 1 = yes). If the answer was yes, they also indicated the extent to which the symptom disturbed them on a 5-point scale (1 = not disturbing, 5 = very disturbing). For each symptom, we recoded responses into a 6-point symptom severity score ranging from 0 (no symptom) to 5 (very disturbing symptom) and used these scores in further analyses.

### 2.3 Statistical analyses

JASP software (43) was used for the statistical analyses of the data. Due to missing data, we applied listwise deletion in the analyses and provided the actual sample size by analysis. We used exploratory principal components analysis and confirmatory factor analysis to elaborate on the structure of the symptom severity scores for further analysis. Subsequently, we applied structural equation modeling to examine the latent structure of the variables in the proposed model and the hypothesized paths between latent variables. Model fit was evaluated using standard indices, including the chi-square statistic (χ²), the Comparative Fit Index (CFI), the Tucker–Lewis Index (TLI), and the Root Mean Square Error of Approximation (RMSEA). Model adequacy was assessed in line with widely applied cutoff recommendations (e.g., CFI and TLI ≥.90/.95; RMSEA ≤.08/.06), following Hu and Bentler’s criteria (44). These indices and decision rules are consistent with current practice in psychiatric and psychosomatic research. Standardized regression coefficients (β) were used to quantify the strength of associations (0.10 was considered small, 0.30 moderate, and 0.50 large).

We applied two extensions of the primary path model to ascertain its validity. Latent variables and their relationship were regressed on a series of background variables. The following variables were examined: gender, age, education (recoded into primary, secondary, and higher education), cohabitation with partner (yes/no), the time elapsed since the onset of illness, and subjective financial status. For education, we used dummy coding. First, we regressed the theoretical model’s latent variables on the background variables to identify the significant predictive relationships. Next, the path model of the latent variables was controlled for the background variables.

Moreover, we tested the equivalence of the path model in three multigroup analyses. We employed a stepwise method in these multigroup analyses to constrain the models’ parameters. First, we allowed all parameters to vary freely within the subgroups; then, we gradually constrained factor loadings, intercepts, residual variances and covariances, factor means and variances, and regression coefficients to be equal across the groups. In each step, the changes of χ² and df were tested against the nested model. Moreover, changes in CFI were examined, and a delta CFI > 0.01 was considered an indicator of non-equivalence (45). In the final decision on equivalence, we interpreted an indicator of non-equivalence when a change in χ² was significant, and a change in CFI was greater than 0.01.

## 3 Results

### 3.1 Preliminary analysis

#### 3.1.1 Assumption checks for structural equation modeling

We assessed the observed variables for univariate distributions, particularly for normality based on skewness and kurtosis. All values were within the acceptable thresholds of widely used guidelines (e.g., ± 2 for skewness and ±7 for kurtosis; 46), suggesting that the data reasonably approximated normality. Moreover, given the relatively large sample size (N > 360), we evaluated the structural models using maximum likelihood (ML) estimation (47) and examined the robustness of the results by re-estimating the models with robust maximum likelihood (MLR). This approach allowed us to assess whether potential deviations from multivariate normality had a substantial effect on parameter estimates or model fit. Finally, multicollinearity was assessed by inspecting the intercorrelations among the observed variables. None of the intercorrelations exceeded |0.8|, indicating that even variables belonging to the same (latent) constructs showed adequate empirical distinctiveness.

#### 3.1.2 Structural analysis of bodily symptoms

Using a random half-split sampling design, we performed an exploratory principal component analysis of symptom severity scores to identify the symptom structure for subsequent structural equation models in the first subsample. Based on a parallel analysis, we identified two principal components that explained 20.4% and 19.4% of the variance. The first component represented inflammatory bowel disease-related symptoms (e.g., stomachache, fatigue), while the second represented general symptoms (c.f., **Supplementary Material S1**). The subsequent confirmatory factor analyses in the second random subsample showed that this solution with two correlated latent factors had only moderate fit (χ2 = 145.917; df = 73; p < 0.001; CFI = 0.89; NFI = 0.81; TLI = 0.87; RMSEA = 0.069 (LO90 = 0.05, HI90 = 0.08). We also tested a model with one overarching latent factor for all symptoms, which had limited fit to the data (χ2 = 164.03; df = 74; p < 0.001; CFI = 0.87; NFI = 0.79; TLI = 0.84; RMSEA = 0.076 (LO90 = 0.06, HI90 = 0.09). In contrast, CFA of the reduced set of seven symptoms of the first factor provided acceptable fit (χ2 = 17.29; df = 13; p < 0.186; CFI = 0.98; NFI = 0.95; TLI = 0.98; RMSEA = 0.04 (LO90 = 0.00, HI90 = 0.08). Important to note that all models included a covariance between Symptoms 2 and 3, both of which represent pain in the movement system. These reflect one of the most common non-abdominal complications of inflammatory bowel disease, which 30-50% of patients experience, i.e., joint pain (2). Considering the psychometric and conceptual aspects of the result, we retained the first factor of IBD-related symptoms for further analyses (detailed factor loadings are presented in **Supplementary Material S2**).

### 3.2 Mediation analyses

**Figure 2:**
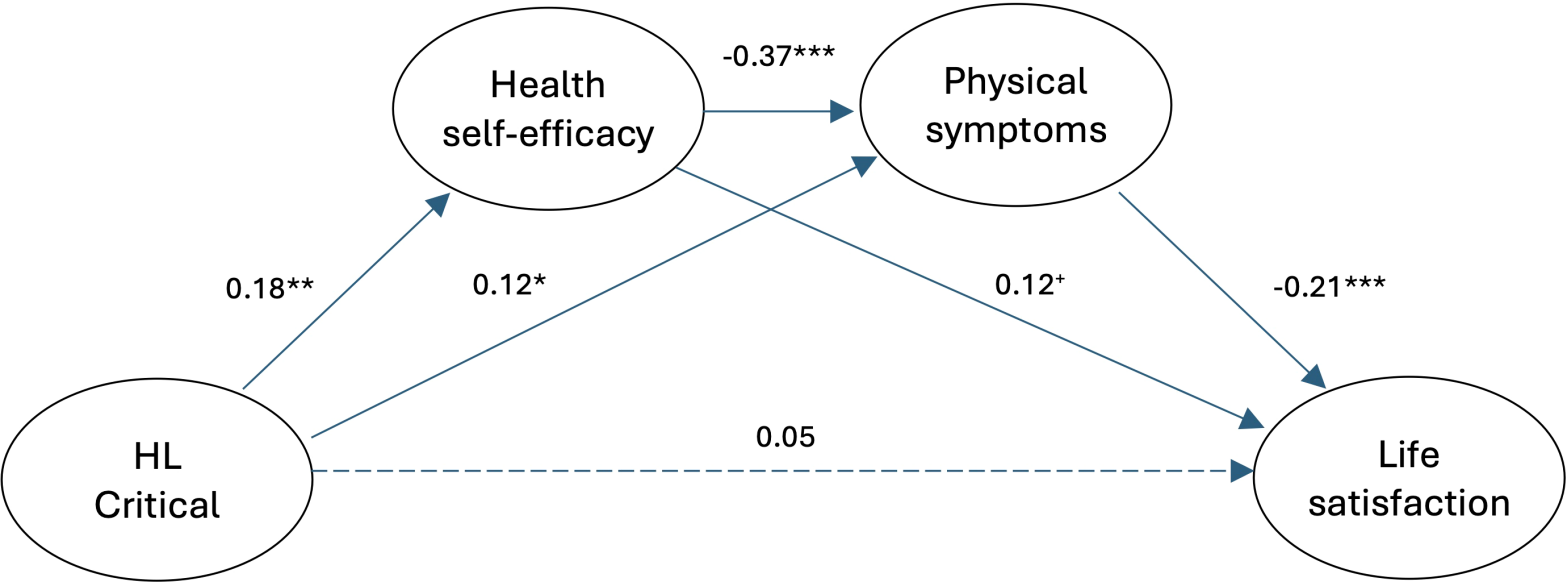
The final model for the whole sample. HL, Health literacy. Manifest variables are not included in the figure. Path coefficients are standardized estimates and are controlled for gender, age, education, time since the diagnosis, relationship status, and financial status. *** p < 0.001, ** p < 0.01, * p < 0.05, + p = 0.062.

We tested the full model in SEM analysis to investigate the associations between the study variables (**Figure 1**). While the model showed acceptable fit (N = 365; χ2 = 623.4; df = 361; p < 0.001; CFI = 0.93; NFI = 0.85; TLI = 0.92; RMSEA = 0.045, 90%CI = 0.039 - 0.050), only critical health literacy was a significant positive predictor of health self-efficacy in this first model (**Supplementary Material S3**). Therefore, we tested a more parsimonious model which included only critical health literacy (c.f., **Figure 2**, for overview), providing a good fit for the data (N = 381; χ2 = 283.8; df = 163; p <.001; CFI = 0.95; NFI = 0.90; TLI = 0.94; RMSEA = 0.044, 90%CI = 0.035 - 0.053). In this model (**Table 2**, upper panel), critical health literacy positively predicted health self-efficacy (β = 0.18, p = .001), and health self-efficacy negatively predicted symptom severity (β = -0.38, p <.001). Moreover, symptom severity was negatively associated with life satisfaction (β = -0.23, p <.001). Furthermore, health self-efficacy positively predicted satisfaction with life (β = 0.30, p <.001). Further information on the measurement model’s coefficients is presented in **Supplementary Material S4**. We additionally re-estimated the models using robust maximum likelihood estimation. Comparison of the fit indices and parameter estimates indicated only minor differences, with no meaningful impact on the overall pattern of results.

**Table 2:** Final model coefficients - baseline and controlled for the background variables.

Baseline model				95% Confidence interval	
Predictor	Outcome	Std. estimate	p	Lower	Upper
Uncontrolled model					
HL-Cr	H-SE	0.19	0.001	0.07	0.30
	Symptoms	0.10	0.099	-0.02	0.22
	SWL	0.04	0.46	-0.07	0.15
H-SE	Symptoms	-0.38	<.001	-0.49	-0.26
	SWL	0.23	<.001	0.10	0.35
Symptoms	SWL	-0.23	<.001	-0.36	-0.11
Controlled for background variables					
HL-Cr	H-SE	0.18	0.001	0.07	0.29
	Symptoms	0.12	0.041	0.01	0.24
	SWL	0.05	0.308	-0.05	0.16
H-SE	Symptoms	-0.37	<.001	-0.49	-0.25
	SWL	0.12	0.062	-0.01	0.24
Symptoms	SWL	-0.21	<.001	-0.34	-0.09

N = 381 and 365 for baseline and controlled models, respectively.

The values in the table are the standardized coefficients.

HL-Cr, health literacy – critical subscale; H-SE, health self-efficacy; SWL, satisfaction with life.

Background variables in the model: gender, age, education, time since the diagnosis, relationship status, and financial status.

When decomposing the indirect effects in the model (**Table 3**), critical health literacy negatively predicted symptom severity (β = -.07, p = .005). This association suggests that higher critical health literacy is associated with fewer symptoms. Critical health literacy also positively predicted satisfaction with life (β = 0.02, p = .026), with health self-efficacy and symptom severity mediating this relationship. Furthermore, health self-efficacy positively predicted satisfaction with life (β = 0.09, p = .002), with symptom severity as a mediator, indicating that individuals with higher health self-efficacy tend to report higher satisfaction with life. While partly low in magnitude, these indirect effects provide evidence of the potential role of critical health literacy in shaping the experiences of patients living with IBD. We also need to consider that indirect effects are often lower than direct effects, and that standard cutoff criteria may not apply when interpreting them (c.f., 48).

**Table 3:** Indirect effects of the mediation model.

Indirect path	Std. Estimate	p	95% CI	
			Lower	Upper
HL-cr → H-SE → Symptoms	-0.07	0.005	-0.119	-0.021
HL-cr → H-SE → Symptoms → SWL	0.02	0.026	0.002	0.031
HL-cr → H-SE → SWL	0.04	0.170	0.008	0.078
HL-cr → Symptoms → SWL	-0.02	0.134	-0.054	0.007
H-SE → Symptoms → SWL	0.09	0.002	0.033	0.143

HL-Cr, health literacy – critical subscale; H-SE, health self-efficacy; SWL, satisfaction with life.

Moreover, we tested for possible confounding effects of background variables, including gender, age, and level of education. We regressed the model’s latent variables on these background variables to identify significant predictive associations. First, we tested a full-prediction model in which each psychological latent variable was regressed on all background variables. The model’s fit was acceptable (χ2 = 496.8; df = 276; p < 0.001; CFI = 0.92; NFI = 0.83; TLI = 0.90; RMSEA = 0.048 (LO90 = 0.048, HI90 = 0.0.41; see **Supplementary Material S5** for the detailed results).

In the second model, the mediation model’s variables were predicted solely by selected background variables that were predictive of the psychological latent variables. The model’s fit was acceptable (N = 352; χ2 = 453.1; df = 272; p < 0.001; CFI = 0.93; NFI = 0.84; TLI = 0.92; RMSEA = 0.043, 90%CI = 0.036 - 0.050). Inspecting the results, we can conclude that the basic pattern of the associations between the latent variables remained similar to that found in the baseline model (see **Figure 2** and **Table 2**, lower panel, for comparison). However, in this controlled model, critical health literacy predicted symptom severity at the p < 0.05 level (β = 0.12, p = .041), and health self-efficacy predicted satisfaction with life only marginally significantly (β = 0.12, p = .062).

### 3.3 Multigroup analyses

As noted in the Statistical Analyses section, we employed a stepwise method to constrain the models’ parameters. In all multigroup analyses, the results indicated equivalence of intercepts but not equivalence of the parameters in subsequent steps. Therefore, we calculated the final models by constraining the factor loadings and intercepts to be equivalent across the two groups. Detailed results of the stepwise constraint procedure are presented in **Supplementary Materials S6a-c**, and path coefficients are presented in **Table 4**.

**Table 4:** Path coefficients in multigroup models.

Predictor	Outcome	Low depression				Elevated depression			
		Std. estimate	SE	z	p	Std. estimate	SE	z	p
HL-Cr	H-SE	0.10	0.08	1.32	0.188	0.39	0.09	4.16	<0.001
	Symptoms	0.12	0.08	1.58	0.114	0.10	0.12	0.81	0.416
	SWL	0.06	0.08	0.77	0.442	0.01	0.11	0.12	0.904
H-SE	Symptoms	-0.40	0.08	-5.22	<0.001	-0.28	0.12	-2.35	0.019
	SWL	0.14	0.09	1.59	0.113	0.29	0.12	2.54	0.011
Symptoms	SWL	-0.19	0.09	-2.05	0.041	-0.12	0.11	-1.09	0.276
		Remission				Relapse			
Predictor	Outcome	Std, estimate	SE	z-value	p	Std, estimate	SE	z-value	p
HL-Cr	H-SE	0.16	0.07	2.27	0.023	0.38	0.11	3.34	0.001
	Symptoms	0.04	0.07	0.56	0.573	0.45	0.13	3.38	0.001
	SWL	-0.02	0.07	-0.27	0.791	0.06	0.16	0.40	0.689
H-SE	Symptoms	-0.29	0.07	-3.96	<0.001	-0.57	0.13	-4.43	<0.001
	SWL	0.21	0.07	2.80	0.005	0.33	0.17	1.91	0.056
Symptoms	SWL	-0.20	0.08	-2.56	0.010	-0.08	0.17	-0.48	0.631

N = 360 and 359 for depression and illness status, respectively.

The values in the table are the standardized coefficients.

HL-Cr, health literacy – critical subscale; H-SE, health self-efficacy; SWL, satisfaction with life.

No results are presented for the multigroup analysis of Crohn’s and colitis ulcerosa, because the subgroups were found equal.

#### 3.3.1 Depression level

We performed multigroup analysis consisting of two levels of depressive symptomatology, which was dummy coded, 0 representing the group of individuals with low levels of depression and 1 representing the group with elevated levels of depression (N = 236 and 124, respectively, using median split). Inspection of changes in fit indices suggested metric invariance, whereas the structural model may differ across the two subgroups (see **Supplementary Material S6a** for a detailed overview). Therefore, we tested the model with factor loadings, intercepts, residuals, and residual covariances constrained to be equal. The model fit was adequate (N = 360; χ2 = 552.9; df = 379; p <.001; CFI = 0.93; TLI = 0.93; RMSEA = 0.050). We describe only the significant paths here, but note that the path between HL-critical and health self-efficacy was not significant (β = 0.10, p = 0.19) in the low-depression group. In contrast, self-efficacy negatively predicted the IBD-relevant symptoms (β = -0.40, p <.001), and higher symptom severity predicted lower satisfaction with life (β = -0.19, p = 0.041). In the group with elevated depression, critical health literacy positively predicted health self-efficacy (β = 0.39, p <.001), which, in turn, negatively predicted IBD-relevant symptoms (β = -0.28, p = .002) and positively predicted satisfaction with life (β = 0.29, p = .011).

#### 3.3.2 Disease status

We performed a second multigroup analysis with two groups: respondents in remission (N = 278) and relapse (N = 81). Inspection of changes in fit indices suggested metric invariance, whereas the structural model may differ across the two subgroups (see **Supplementary Material S6b** for a detailed overview). Therefore, we tested the model with factor loadings, intercepts, residuals, and residual covariances constrained to be equal. The model fit was adequate (N = 359; χ2 = 593.8; df = 379; p <.001; CFI = 0.91; TLI = 0.91; RMSEA = 0.056). We describe only the significant paths here. In the remission group, health self-efficacy was positively predicted by critical health literacy (β = 0.16, p = 0.024). In contrast, self-efficacy negatively predicted IBD-relevant symptoms (β = -0.29, p <.001), and satisfaction with life positively predicted them (β = 0.21, p = .005). More frequent symptoms predicted lower satisfaction with life (β = -0.20, p = 0.01). In the relapse group, critical health literacy positively predicted health self-efficacy (β = 0.38, p <.001); however, it also predicted symptom severity (β = 0.45, p <.001). Higher self-efficacy predicted lower symptom severity and higher satisfaction with life (β = -0.56, p <.001, and β = 0.33, p = .056, respectively).

#### 3.3.3 Illness type

We performed a third multigroup analysis with two separate groups: respondents living with Crohn’s disease (N = 236) and colitis ulcerosa (N = 125). The changes in model fit did not indicate a significant difference between the nested models (see **Supplementary Material S6c** for a detailed overview). The fit of the final model with all indicators constrained to be equal was acceptable (N = 359; χ2 = 580.5; df = 390; p< 0.001; CFI = .93; TLI = .93; RMSEA = .052). In line with the results, we may assume that the baseline model, as presented in section 3.1, equally applies for the respondents with Crohn’s disease and colitis ulcerosa.

## 4 Discussion

We aimed to depict the complex relationships among depression, health literacy, and health self-efficacy and their effects on patient-reported outcomes in a larger sample of patients with inflammatory bowel disease. Since one strain of previous research indicates a solid association between inflammatory bowel disease, mental health, and quality of life (8, 17) and another one points out health literacy and health self-efficacy as effective patient-related skills to manage the disease (3, 29, 30), we built up sequential equation models to examine mediating and moderating effects among these variables. The originality of our analysis lies in treating the bidirectional relation between health literacy and depression as a cornerstone and, beyond sequential equation models, in conducting multigroup analyses that carefully examine the role of health literacy at different levels of depression.

According to the results, only one type of health literacy, namely critical health literacy, played a significant role in predicting health self-efficacy of patients with inflammatory bowel disease, which, in turn, predicted lower perceived symptom severity and higher life satisfaction. This result aligns with previous ones measuring different types of health literacy and patient-reported outcomes in patients with chronic obstructive pulmonary disease (49). Specifically, more basic types of health literacy, such as functional skills, did not contribute to the empowerment of patients or their subjective health status. One possible reason for the nonsignificance of functional and communicative HL in our study might lie in their nature: these skills enable patients to acquire and understand health-related information; however, they do not necessarily enable them to apply that information to personal circumstances or make health-related decisions.

Our full SEM model also reflects that higher critical health literacy is associated with lower symptom severity. It suggests that applying relevant disease-specific information in a more personalized way to manage a chronic disease may help reduce symptom severity. However, the finding that critical health literacy did not directly predict satisfaction with life suggests that more complex forms of health literacy also operate only through changes in health self-efficacy and symptom severity. This association is in line with Hu and Xu’s (30) findings, which confirmed a chain mediating effect between fear of progression, health literacy, self-care, and health-related quality of life of patients with inflammatory bowel disease.

Unlike critical health literacy, health self-efficacy predicted both directly and, through symptom severity, indirectly satisfaction with life. A previous study found that self-efficacy is more decisive than health literacy in patient-reported outcomes, specifically among patients with insulin-treated T2DM (50).

The similar mediating effects among critical health literacy, health self-efficacy, symptom severity, and satisfaction with life were confirmed in the present study after controlling for a series of sociodemographic and illness-related background variables, which gives a unique role of critical health literacy in managing IBD.

Taking the role of depression into account in multigroup analyses, we found a clear difference between the presence of depressive symptoms in inflammatory bowel disease and the lack of this comorbidity: critical health literacy predicted health self-efficacy in the previous group, whereas it did not exert any effect in the low depression group. This association means that knowing the progression and the possible complications of inflammatory bowel disease (i.e., depression as a comorbid state) makes them more manageable in their presence.

When comparing the models across remission and relapse stages of the disease, we found the above-presented patterns in the interrelatedness of the variables for patients in remission. However, in the acute state of the disease, a new association emerged: higher critical health literacy also predicted higher symptom severity. This difference points to the higher sensitivity of relapse periods in terms of disease-related attention and emotions. In our understanding, those who are able to make good use of information about their disease are more likely to feel the severity of the symptoms during relapse periods and attribute them to IBD. Previous studies have also highlighted the increased need for providing support in effective disease self-management during relapse periods (21).

To the best of our knowledge, this is the first research examining depression, health literacy, health self-efficacy, and IBD-related symptoms in such a complex model with a large sample of patients with inflammatory bowel disease. Our findings indicate that only critical health literacy has a potential predictive effect in a mediation model, and its more prevalent role in the presence of elevated levels of depression is considered to be an original contribution to understanding the necessary skills of maintaining a satisfactory life for patients with inflammatory bowel disease. While the present study focused on intrapersonal characteristics, future research should also examine how interpersonal and social-contextual factors—such as support from relatives and healthcare professionals—shape health literacy and its association with patient outcomes.

### 4.1 Limitations

Due to our study’s cross-sectional nature, we cannot explore causal relationships among our variables. In the mediational analysis, we assumed that the tested associations had specific directions; however, this implies prediction, not causation. This highlights the need for future longitudinal research in this field. Moreover, we used only patient-reported data in the test battery and could not rely on medical records. Importantly, self-report data on symptoms and subjective states are prone to certain types of bias (c.f., 51, 52). The potentially high proportion of biased responses may also explain the relatively low proportion of explained variance in the PCA of the somatic symptoms. The additional use of health-care-professional-provided data on depression or illness-related variables would have increased the reliability of our data. For certain key constructs, such as depression and health self-efficacy, we used short versions of the original scales, which may reduce construct validity. Specifically, a short version of DASS enabled only a median split in the sample for depressivity. Future studies using more comprehensive depression assessments may allow the application of clinical cut-off scores, enabling a more fine-grained examination of subgroup differences.

Although multiple associations reached statistical significance, the magnitude of some effects—particularly indirect effects—was modest, which limits their practical implications. These findings should therefore be interpreted primarily as evidence of underlying associations rather than as indicators of substantial clinical impact.

Finally, our data collection was carried out by a single Hungarian clinical center. Although this center provides nationwide healthcare services, reliance on one center limits the generalizability of the findings to other countries, given potential cultural or healthcare system differences. Relying on a single center also affected the sample size and composition. For example, participants in a relapse condition represented only about one-fifth of the total sample; therefore, the multigroup analyses for this subgroup may lack generalizability. In the future, more targeted studies should focus on a more fine-grained assessment of the characteristics of this study.

### 4.2 Practical implications

Managing a complex chronic disease like IBD requires multiple skills from the patients. According to the present results, providing information alone is not enough to increase health self-efficacy; the highest level of health literacy might be achieved through critical analysis of information about the disease, its application to the patient’s personal needs, and understanding of the functioning of the healthcare system and available services. By focusing on this and fostering a sense of efficacy in patients in health-related fields, successful patient education can be planned. Besides, the consistent negative association between symptom severity and life satisfaction reinforces the clinical relevance of symptom management. A practical, adaptable, and autonomous knowledge about IBD is even more important when comorbidities (e.g., depression), sensitive periods of the disease (i.e., relapse) are present. Clinicians, patient educators, or policymakers should consider focusing on developing the highest level of health literacy and patient self-efficacy among patients living with IBD, as well as tailoring targeted interventions, especially for vulnerable subpopulations.

### 4.2 Conclusion

The study underscores the importance of critical health literacy and self-efficacy in IBD self-management. These skills are not mere technical add-ons—they are central resources in managing illness experience and quality of life. Based on the results, we suggest that patient education today should foster critical thinking and give voice to patient experiences as part of a higher level of health literacy, especially in the presence of mental comorbidities. Depression and symptom burden during relapses are not just outcomes but shaping forces in how illness knowledge and agency translate into lived experience.

## Data Availability

The database and analyses can be retrieved from https://osf.io/du5y8/files/osfstorage.
