# Supplementary material for: Is (critical) health literacy a key to better psychosomatic functioning in patients with inflammatory bowel disease? Testing a mediation model
Source: Front Psychiatry. 2026 Feb 6;17:1643641. doi: 10.3389/fpsyt.2026.1643641 (PMC12920207; doi:10.3389/fpsyt.2026.1643641)
Supplement: Supplementary file 1 [file Table1.docx]

# Supplement S1.

Supplement S1. Principal Component Loadings of Symptom Severity Items

|  |  | PC1 | PC2 | Uniqueness |
| --- | --- | --- | --- | --- |
| Symptom_1 | Abdominal pain | 0.59 |  | 0.63 |
| Symptom_2 | Waist or back pain | 0.51 |  | 0.75 |
| Symptom_3 | Arm, leg or joint pain such as knee, hip etc. | 0.59 |  | 0.60 |
| Symptom_4 | Headache |  | 0.39 | 0.80 |
| Symptom_5 | Chest pain |  | 0.64 | 0.64 |
| Symptom_6 | Dizziness |  | 0.59 | 0.46 |
| Symptom_7 | Feeling of fainting, faintness |  | 0.56 | 0.51 |
| Symptom_8 | Strong or rapid palpitations |  | 0.76 | 0.44 |
| Symptom_9 | Dyspnoea, shortness of breath |  | 0.69 | 0.50 |
| Symptom_10 | Pain or problems during sexual intercourse |  | 0.49 | 0.78 |
| Symptom_11 | Constipation, dilute or too frequent stools | 0.66 |  | 0.56 |
| Symptom_12 | Nausea, bloating, intestinal gas, digestive problems | 0.77 |  | 0.43 |
| Symptom_13 | Fatigue, lack of energy | 0.60 |  | 0.58 |
| Symptom_14 | Sleep-related problems | 0.38 |  | 0.72 |

Notes: N= 208
Principal component analysis with oblimin rotation
Only component loading over |.4| are presented
