# Supplementary material for: Is (critical) health literacy a key to better psychosomatic functioning in patients with inflammatory bowel disease? Testing a mediation model
Source: Front Psychiatry. 2026 Feb 6;17:1643641. doi: 10.3389/fpsyt.2026.1643641 (PMC12920207; doi:10.3389/fpsyt.2026.1643641)
Supplement: Supplementary file 2 [file Table2.docx]

# Supplement S2.

Supplement S2. Confirmatory factor analysis for IBD-related symptoms

|  |  |  |  | 95% Confidence interval | |
| --- | --- | --- | --- | --- | --- |
| Latent | Indicator | Std. estimate | p | Lower | Upper |
| Symptoms | Abdominal pain | 0.54 | < .001 | 0.45 | 0.62 |
|  | Waist or back pain | 0.37 | < .001 | 0.27 | 0.47 |
|  | Arm, leg or joint pain such as knee, hip etc. | 0.43 | < .001 | 0.34 | 0.53 |
|  | Constipation, dilute or too frequent stools | 0.62 | < .001 | 0.54 | 0.70 |
|  | Nausea, bloating, intestinal gas, digestive problems | 0.73 | < .001 | 0.66 | 0.79 |
|  | Fatigue, lack of energy | 0.56 | < .001 | 0.47 | 0.64 |
|  | Sleep-related problems | 0.45 | < .001 | 0.36 | 0.55 |

Notes: N= 207

Including a structural covariance between Symptom_2 and Symptom_3 (st. estimate: 0.39, p < .001)
