# Supplementary material for: Is (critical) health literacy a key to better psychosomatic functioning in patients with inflammatory bowel disease? Testing a mediation model
Source: Front Psychiatry. 2026 Feb 6;17:1643641. doi: 10.3389/fpsyt.2026.1643641 (PMC12920207; doi:10.3389/fpsyt.2026.1643641)
Supplement: Supplementary file 3 [file Table3.docx]

# Supplement S3.

Supplement S3. Full model coefficients

| Regression weights | |  |  |  | |
| --- | --- | --- | --- | --- | --- |
|  |  |  |  | 95% Confidence interval | |
| Predictor | Outcome | Std. estimate | p | Lower | Upper |
| HL-Func | H-SE | -0.004 | 0.950 | -0.13 | 0.12 |
|  | Symptoms | 0.05 | 0.407 | -0.07 | 0.18 |
|  | SWL | -0.10 | 0.081 | -0.22 | 0.01 |
| HL-Comm | H-SE | -0.09 | 0.458 | -0.33 | 0.15 |
|  | Symptoms | 0.13 | 0.302 | -0.11 | 0.36 |
|  | SWL | 0.09 | 0.414 | -0.13 | 0.31 |
| HL-Cr | H-SE | 0.26 | 0.020 | 0.04 | 0.48 |
|  | Symptoms | 0.004 | 0.970 | -0.22 | 0.23 |
|  | SWL | -0.01 | 0.896 | -0.22 | 0.19 |
| H-SE | Symptoms | -0.37 | < 0.001 | -0.49 | -0.25 |
|  | SWL | 0.24 | < 0.001 | 0.12 | 0.37 |
| Symptoms | SWL | -0.22 | 0.001 | -0.35 | -0.09 |
|  | |  |  |  |  |
| Factor covariances | |  |  |  |  |
| HL-Func | HL-Comm | -0.12 | 0.059 | -0.25 | 0.01 |
| HL-Func | HL-Cr | 0.00 | 0.990 | 1-0.12 | 0.12 |
| HL-Comm | HL-Cr | 0.74 | < 0.001 | 0.67 | 0.82 |

Notes:

The model is represented in Figure 1

HL-Func, health literacy – functional subscale; HL-Comm, health literacy – communicative subscale; HL-Cr, health literacy – critical subscale; H-SE, health self-efficacy; SWL, satisfaction with life; Symptoms, IBD relevant symptoms
