# Supplementary material for: Is (critical) health literacy a key to better psychosomatic functioning in patients with inflammatory bowel disease? Testing a mediation model
Source: Front Psychiatry. 2026 Feb 6;17:1643641. doi: 10.3389/fpsyt.2026.1643641 (PMC12920207; doi:10.3389/fpsyt.2026.1643641)
Supplement: Supplementary file 4 [file Table4.docx]

# Supplement S4.

Supplement S4. Factor loadings of the final measurement model

|  | | | | | 95% Confidence interval | |
| --- | --- | --- | --- | --- | --- | --- |
| Latent | | Indicator | Std. estimate | p | Lower | Upper |
| HL-Cr |  | FCCHL_11 | 0.75 | < .001 | 0.70 | 0.84 |
|  |  | FCCHL_12 | 0.92 | < .001 | 0.88 | 0.96 |
|  |  | FCCHL_13 | 0.72 | < .001 | 0.66 | 0.78 |
|  |  | FCCHL_14 | 0.64 | < .001 | 0.58 | 0.71 |
| H-SE |  | SE_1 | 0.67 | < .001 | 0.60 | 0.74 |
|  |  | SE_2 | 0.69 | < .001 | 0.62 | 0.75 |
|  |  | SE_3 | 0.79 | < .001 | 0.73 | 0.84 |
|  |  | SE_4 | 0.76 | < .001 | 0.70 | 0.81 |
| SWL |  | SWL_1 | 0.79 | < .001 | 0.75 | 0.84 |
|  |  | SWL_2 | 0.74 | < .001 | 0.69 | 0.79 |
|  |  | SWL_3 | 0.85 | < .001 | 0.81 | 0.89 |
|  |  | SWL_4 | 0.72 | < .001 | 0.66 | 0.78 |
|  |  | SWL_5 | 0.60 | < .001 | 0.52 | 0.67 |
| Symptoms |  | Symptom_1 | 0.54 | < .001 | 0.45 | 0.63 |
|  |  | Symptom_2 | 0.38 | < .001 | 0.27 | 0.48 |
|  |  | Symptom_3 | 0.44 | < .001 | 0.34 | 0.53 |
|  |  | Symptom_11 | 0.62 | < .001 | 0.54 | 0.69 |
|  |  | Symptom_12 | 0.73 | < .001 | 0.65 | 0.79 |
|  |  | Symptom_13 | 0.56 | < .001 | 0.47 | 0.67 |
|  |  | Symptom_14 | 0.45 | < .001 | 0.36 | 0.55 |
| Notes: N = 381  HL-Cr, health literacy – critical subscale; H-SE, health self-efficacy; SWL, satisfaction with life; Symptoms, IBD relevant symptoms | | | | | | |
