# Supplementary material for: Is (critical) health literacy a key to better psychosomatic functioning in patients with inflammatory bowel disease? Testing a mediation model
Source: Front Psychiatry. 2026 Feb 6;17:1643641. doi: 10.3389/fpsyt.2026.1643641 (PMC12920207; doi:10.3389/fpsyt.2026.1643641)
Supplement: Supplementary file 5 [file Table5.docx]

# Supplement S5.

Supplement S5. Prediction of the model’s latent variables by the background characteristics

|  |  |  |  | 95% Confidence interval | |
| --- | --- | --- | --- | --- | --- |
| Outcome | Predictor | Std.  estimate | p | Lower | Upper |
| HL-Cr | gender (female vs. male) | 0.12 | 0.03 | 0.02 | 0.23 |
|  | age (years) | 0.21 | 0.00 | 0.09 | 0.34 |
|  | education (secondary vs. primary) | -0.13 | 0.07 | -0.27 | 0.01 |
|  | education (higher vs. primary) | 0.26 | 0.00 | 0.12 | 0.41 |
|  | relationship (yes vs. no) | -0.01 | 0.89 | -0.12 | 0.1 |
|  | diagnosis time (years) | -0.16 | 0.01 | -0.28 | -0.04 |
|  | subjective financial status | 0.03 | 0.64 | -0.09 | 0.14 |
| SE | gender (female vs. male) | -0.05 | 0.43 | -0.16 | 0.07 |
|  | age (years) | 0.07 | 0.35 | -0.07 | 0.20 |
|  | education (secondary vs. primary) | 0.03 | 0.69 | -0.12 | 0.18 |
|  | education (higher vs. primary) | -0.08 | 0.30 | -0.24 | 0.07 |
|  | relationship (yes vs. no) | -0.03 | 0.61 | -0.15 | 0.09 |
|  | diagnosis time (years) | 0.01 | 0.86 | -0.12 | 0.15 |
|  | subjective financial status | 0.22 | 0.00 | 0.11 | 0.34 |
| Symptoms | gender (female vs. male) | 0.22 | 0.00 | 0.11 | 0.33 |
|  | age (years) | 0.12 | 0.09 | -0.02 | 0.26 |
|  | education (secondary vs. primary) | 0.07 | 0.35 | -0.08 | 0.22 |
|  | education (higher vs. primary) | -0.22 | 0.00 | -0.37 | -0.07 |
|  | relationship (yes vs. no) | -0.12 | 0.04 | -0.23 | 0.00 |
|  | diagnosis time (years) | 0.02 | 0.78 | -0.11 | 0.15 |
|  | subjective financial status | -0.15 | 0.02 | -0.27 | -0.03 |
| SWL | gender (female vs. male) | -0.02 | 0.71 | -0.12 | 0.08 |
|  | age (years) | -0.08 | 0.18 | -0.2 | 0.04 |
|  | education (secondary vs. primary) | -0.09 | 0.17 | -0.22 | 0.04 |
|  | education (higher vs. primary) | 0.00 | 1.00 | -0.13 | 0.13 |
|  | relationship (yes vs. no) | -0.22 | 0.00 | -0.32 | -0.12 |
|  | diagnosis time (years) | -0.03 | 0.60 | -0.14 | 0.08 |
|  | subjective financial status | 0.45 | 0.00 | 0.36 | 0.54 |

Notes: N = 352

HL-Cr, health literacy – critical subscale; H-SE, health self-efficacy; SWLS, satisfaction with life
