# Supplementary material for: Is (critical) health literacy a key to better psychosomatic functioning in patients with inflammatory bowel disease? Testing a mediation model
Source: Front Psychiatry. 2026 Feb 6;17:1643641. doi: 10.3389/fpsyt.2026.1643641 (PMC12920207; doi:10.3389/fpsyt.2026.1643641)
Supplement: Supplementary file 6 [file Table6.docx]

# Supplement S6.

Supplement S6a. Stepwise constraint procedure, depression level

|  | χ² | df | p | CFI | TLI | RMSEA | delta χ² | delta df | p |
| --- | --- | --- | --- | --- | --- | --- | --- | --- | --- |
| Free | 468.1 | 326 | < .001 | 0.94 | 0.93 | 0.05 |  |  |  |
| Loadings | 478.7 | 342 | < .001 | 0.94 | 0.93 | 0.04 | 10.6 | 16 | 0.83 |
| Intercepts | 500.1 | 354 | < .001 | 0.93 | 0.93 | 0.05 | 21.3 | 16 | 0.17 |
| Residuals | 552.6 | 374 | < .001 | 0.92 | 0.92 | 0.05 | 52.6 | 20 | < .001 |
| Residual covariances | 552.9 | 375 | < .001 | 0.92 | 0.92 | 0.04 | 0.3 | 1 | 0.58 |
| Means | 609.7 | 383 | < .001 | 0.90 | 0.90 | 0.05 | 56.8 | 4 | < .001 |
| Regressions | 621 | 389 | < .001 | 0.90 | 0.90 | 0.06 | 11,3 | 6 | 0,08 |

Supplement S6b. Stepwise constraint procedure, illness status

|  | χ² | df | p | CFI | TLI | RMSEA | delta χ² | delta df | p |
| --- | --- | --- | --- | --- | --- | --- | --- | --- | --- |
| Free | 507.9 | 326 | < .001 | 0.92 | 0.91 | 0.05 |  |  |  |
| Loadings | 522.8 | 342 | < .001 | 0.92 | 0.92 | 0.05 | 14.9 | 16 | 0.532 |
| Intercepts | 555.4 | 358 | < .001 | 0.93 | 0.91 | 0.05 | 32.6 | 16 | 0.008 |
| Residuals | 593.4 | 378 | < .001 | 0.91 | 0.91 | 0.05 | 38.0 | 20 | 0.009 |
| Residual covariances | 593.8 | 379 | < .001 | 0.91 | 0.91 | 0.05 | 0.4 | 1 | 0.527 |
| Means | 639.6 | 383 | < .001 | 0.89 | 0.89 | 0.06 | 45.8 | 4 | < 0.001 |
| Regressions | 656.5 | 389 | < .001 | 0.89 | 0.89 | 0.06 | 16.9 | 6 | 0.10 |

Supplement S6c. Stepwise constraint procedure, illness type

|  | χ² | df | p | CFI | TLI | RMSEA | delta χ² | delta df | p |
| --- | --- | --- | --- | --- | --- | --- | --- | --- | --- |
| Free | 503.6 | 328 | < .001 | 0.93 | 0.92 | 0.05 |  |  |  |
| Loadings | 524.7 | 344 | < .001 | 0.93 | 0.92 | 0.05 | 21.1 | 16 | 0.17 |
| Intercepts | 538.5 | 360 | < .001 | 0.93 | 0.93 | 0.05 | 13.8 | 16 | 0.61 |
| Residuals | 568.6 | 380 | < .001 | 0.93 | 0.93 | 0.05 | 30.1 | 20 | 0.07 |
| Residual covariances | 570.4 | 381 | < .001 | 0.92 | 0.92 | 0.05 | 1.8 | 1 | 0.18 |
| Means | 576.4 | 385 | < .001 | 0.92 | 0.92 | 0.05 | 6.0 | 4 | 0.20 |
| Regressions | 580.5 | 390 | < .001 | 0.92 | 0.93 | 0.05 | 4.1 | 5 | 0.54 |
